# Strain-Transcendent Immune Response to Recombinant Var2CSA DBL5-ε Domain Block *P. falciparum* Adhesion to Placenta-Derived BeWo Cells under Flow Conditions

**DOI:** 10.1371/journal.pone.0012558

**Published:** 2010-09-03

**Authors:** Pablo Fernandez, Stéphane Petres, Salaheddine Mécheri, Jürg Gysin, Artur Scherf

**Affiliations:** 1 Unité de Biologie des Interactions Hôte-Parasite, Institut Pasteur, Paris, France; 2 CNRS URA2581, Paris, France; 3 Host Pathogen Interactions Unit, Institut Pasteur, Abymes, France; Federal University of São Paulo, Brazil

## Abstract

**Background:**

Pregnancy-associated malaria (PAM) is a serious consequence of the adhesion to the placental receptor chondroitin sulfate A (CSA) of ***Plasmodium falciparum***-infected erythrocytes (PE) expressing the large cysteine-rich multi-domain protein var2CSA. Women become resistant to PAM, and develop strain-transcending immunity against CSA-binding parasites. The identification of var2CSA regions that could elicit broadly neutralizing and adhesion-blocking antibodies is a key step for the design of prophylactic vaccine strategies.

**Methodology:**

*Escherichia coli* expressed var2CSA DBL domains were refolded and purified prior to immunization of mice and a goat. Protein-G-purified antibodies were tested for their ability to block FCR3^CSA^-infected erythrocytes binding to placental (BeWo) and monkey brain endothelial (ScC2) cell lines using a flow cytoadhesion inhibition assay mimicking closely the physiological conditions present in the placenta at shear stress of 0.05 Pa. DBL5-ε, DBL6-ε and DBL5-6-ε induced cross-reactive antibodies using Alum and Freund as adjuvants, which blocked cytoadhesion at values ranging between 40 to 96% at 0.5 mg IgG per ml. Importantly, antibodies raised against recombinant DBL5-ε from 3 distinct parasites genotypes (HB3, Dd2 and 7G8) showed strain-transcending inhibition ranging from 38 to 64% for the heterologuous FCR3^CSA^.

**Conclusions:**

Using single and double DBL domains from var2CSA and Alum as adjuvant, we identified recombinant subunits inducing an immune response in experimental animals which is able to block efficiently parasite adhesion in a flow cytoadhesion assay that mimics closely the erythrocyte flow in the placenta. These subunits show promising features for inclusion into a vaccine aiming to protect against PAM.

## Introduction

*Plasmodium falciparum*-infected erythrocytes (PE) sequester in the placenta by adhering to chondroitin sulfate A (CSA) expressed on the surface of human syncytiotrophoblasts [1]. The massive sequestration of parasites is believed to cause pregnancy-associated malaria (PAM) in primigravid women, since in multigravid women improved health is linked to highly reduced parasite sequestration [2]. Naturally acquired antibodies protect against PAM as a function of parity through a mechanism that appears to be IgGs blocking binding of PE to CSA [3]–[5]. The VAR2CSA gene product is expressed on the surface of PE selected on CSA and on parasite isolates from infected placentas [6]–[10]. Although several DBL domains are linked to CSA binding [11], the expression of full length recombinant var2CSA (exon 1) suggest that high affinity binding to CSA requires the cooperation of several domains [12], [13]. Disruption of this gene leads to the irreversible loss of binding to CSA, pointing to this molecule as the prime target for intervention strategies for PAM [14]. Two major problems are linked to var2CSA. First, it is a protein with a very large external domain of approximately 300 kDa. Second, although the gene is found in all *P. falciparum* isolates, some regions show considerable genetic diversity [15]. A major ongoing effort in several laboratories is the identification of semi-conserved subunit regions that induce efficiently adhesion-blocking immune response in animals. Several recent publications have reported some progress into this direction [16]–[18]. In these studies, the evaluation of cytoadhesion inhibition has been based on assays using purified non-placental sources of CSA bound to plastic dishes (static assays). This test is a very convenient assay suitable for high throughput screening of sera. It does, however, neither mimic the exact CSA-sulfation pattern and CSA density on placental cells nor the shear stress due to placental blood flow.

In this study, four different DBL5-ε, two different double DBL5-6-ε and one DBL6-ε domains were produced in an *E. coli* expression system. Immunization protocols using Freund's and Alum as adjuvants were used to raise antisera in mice and a goat. We report significant levels of inhibition of parasite adhesion for purified IgG obtained with both adjuvants, using an *in vitro* assay that mimics very closely the physiological situation in the placenta during infection. Purified IgGs from animals immunized with homologous and heterologous var2CSA DBL regions were evaluated for their capacity to block FCR3^CSA^ PEs binding to a placental cell line (BeWo) using a flow cytoadhesion chamber. We report here that domains at the C-terminal region of var2CSA external domain generated high levels of strain-transcending cytoadhesion-blocking antibodies.

## Methods

### Ethics Statement

All animal experiments were approved and conducted in accordance with the Institut Pasteur Biosafety Committee. Animals were maintained in an animal facility which is licensed by the French Ministry of Agriculture (agreement B 75 15-08 dated May 22, 2008). Facility has central air conditioning equipment that maintains constant temperature of 22±2°C. Air is renewed at least 20 times per hour in animal rooms. Fluorescent light is provided with a 12∶12 h light:dark cycle.

In France, review of experimental protocols by an ethic committee is not mandatory. All researchers performing animal experiments in this study were directly responsible for the experimental protocols and obtained individual licenses from the French Ministry of Agriculture.

### 1. Prokaryotic expression, refolding and purification

#### *1.1. Cloning*

Genes encoding FCR3 var2CSA (accession AY372123) DBL5-ε (residues 2003–2281), DBL6-ε (residues 2297–2590) and double DBL5-6-ε (residues 2003–2590), 7G8 var2CSA (accession EF614233) DBL5-ε (residues 2019–2311), Dd2 var2CSA DBL5-ε (residues 2009–2291), and HB3-1 DBL5-ε (residues 2026–2313) and double DBL5-6-ε (residues 2026–2601) domains were cloned into the pET28a expression vector between the NdeI and BamHI restriction sites. FCR3 var2CSA sequences were obtained by PCR over a synthetic gene template encoding for var2CSA DBL5-6-ε, designed with optimized codons for *E. coli* expression, and using the following oligonucleotides:

FCR3-DBL5-6-For, 5′-GAAGGAGATATACATATGCTGATTGCGGATGG-3′; FCR3-DBL6-For, 5′-GACACGGAGATCATATGGCGTTCAAACAAATCAAAGAACAGG-3′; FCR3-DBL5-Rev, 5′-TCCGTCAGGATCCTTAAAAGCCGCACGGGC-3′; FCR3-DBL6-Rev, 5′-TCCGTCAGGATCCTATTCTTTCAGATAATCCGGCGC-3′. The other strains sequences were obtained by PCR over the corresponding genomic DNA template using the following oligonucleotides: 7G8-Dd2-DBL5-For, 5′-GAAGGAGATATACATATGTTAATTGGAGATGC-3′; HB3-DBL5-For, 5′-GAAGGAGATATACATATGTTAATTGCAGATGCTATAGG-3′; 7G8-HB3-DBL5-Rev, 5′-TCCGTCAGGATCCTTAAAATCCACACGGAC-3′; Dd2-DBL5-Rev, 5′-TCCGTCAGGATCCTAAAAACGACATGAAC-3′; HB3-DBL5-6-Rev, 5′-TCCGTCAGGATCCTTATCTTTCAAGTACTCATG-3′.

#### *1.2. Expression*

Transformed *E. coli* BL21 (DE3) cells were grown at 37°C in LB medium with 30 µ g/ml of kanamycin to an absorbance of 0.5 at 600 nm, and were then induced with 1.0 mM isopropyl-β-D-thiogalactopyranoside (IPTG) for 3 h at 37°C under good aeration. For DBL6-ε soluble expression induction was performed with 0.1 mM IPTG, overnight at 20°C. Cells were harvested by centrifugation at 6000 g. The pellets of culture were resuspended in 50 mM Tris.HCl, pH 8.5, containing 150 mM NaCl. The cells were disrupted by sonication on ice and the suspensions were centrifuged for 20 min at 5000 g.

#### *1.3. Refolding and Purification*

FCR3-DBL6-ε was expressed as a soluble protein in the soluble fraction and therefore purified from the supernatant using a HisTrap FF Ni-affinity column. After this single purification step, protein yield (not optimized conditions) was 2.5 mg/liter of culture. For all the other proteins, expressed as insoluble inclusion bodies, pellets were resuspended and washed twice with 30 ml of 50 mM Tris.HCl, pH 8.5, containing 150 mM NaCl and finally centrifuged for 20 min at 10000 g. The pellets containing inclusion bodies were then denatured during 2 h at 25°C under agitation in 50 mM Tris.HCl pH 8.0, containing 200 mM NaCl, 2.0 mM EDTA, 7.0 M GuHCl and 10 mM TCEP. The suspensions were centrifuged for 30 min at 15000 g and the pellet was discarded.

Refolding was assayed with the IFOLD™ Protein Refolding System 2 (Novagen) according to the manufacturer's instructions. Once the best condition was established, each denatured protein was refolded by rapid dilution (1∶50, 5 mg/mL) and maintained with gently shaking overnight. Composition of the refolding solutions were as follow: 50 mM TAPS pH 8.5, 0.5 M NDSB-256, 6.0 mM reduced glutathione, 4.0 mM oxidized glutathione, 240 mM NaCl, and 10 mM KCl (for DBL5-ε domains); 50 mM Hepes pH 7.5, 6.0 mM reduced glutathione, 4.0 mM oxidized glutathione, 240 mM NaCl, and 10 mM KCl (for DBL5-6-ε domains). After refolding, solutions were centrifuged for 30 min at 20000 g to eliminate aggregated proteins. Supernatants were passed on a HisTrap FF Ni-affinity column previously equilibrated with the same buffer, and connected to an FPLC Akta System. Proteins were eluted with an imidazole gradient (0 to 0.5 M) and aliquots containing each purified DBL domain were pooled. After dialysis against PBS and concentration by means of Macro- and Micro-sep concentrators, a further stage of gel filtration (Superdex 75, Amersham Pharmacia Biotech) was required to separate the aggregated material from the monomeric proteins and other impurities. Purified DBLs were subsequently concentrated by means of Macro- and Micro-sep concentrators. Protein concentration was determined using the Bio-Rad protein assay. Purity of the samples was checked by SDS-PAGE and Western blot, under reduced (by adding DTT) or non reduced conditions.

Protein yields (not optimized conditions) after refolding and all purification steps were 3.0 mg/liter of culture for FCR3-DBL5-ε, 0.4 mg/liter of culture for 7G8-DBL5-ε, 0.5 mg/liter of culture for HB3-DBL5-ε, 1.0 mg/liter of culture for Dd2-DBL5-ε, 1.5 mg/liter of culture for FCR3-DBL5-6-ε, and 0.5 mg/liter of culture for HB3-DBL5-6-ε.

### 2. Immunizations

Groups of five Balb/c mice (Charles River) received a primary subcutaneous injection of 20 µg recombinant antigens dissolved in 100 µl of NaCl 0.9% and emulsified at 1∶1 in 100 µl of complete Freund's adjuvant or 100 µl of Alum (Thermo Scientific- Rockford IL USA). Two additional injections were performed at days 14 and 42 after the first injection using the same amount and antigen batches emulsified at 1∶1 in incomplete Freund's adjuvant or in Alum. Mice were bled by orbital sinus puncture one day before the primary antigen injection and each time preceding an antigen boost. Ex-sanguination was performed at day 70 after primary injection. Serum samples were collected after centrifugation of the blood, and decomplemented for 30 min at 56°C. Goat was immunized using primary intramuscular injection of 200 µg refolded recombinant antigen dissolved in 1 ml of NaCl 0.9% and emulsified at 1∶1 in 1 ml of Alum. Two additional injections were performed at days 35 and 70 after the first injection using same amount and antigen batches emulsified at 1∶1 in 1 ml of Alum.

### 3. IgG Purification and depletion

Hitrap protein G (GE, USA) was used according to manufacturer's instructions for IgG purification of mouse or goat plasma. Bound IgG was eluted in 0.5 ml fractions using 100 mM Glycin pH 2.5 (tubes were preloaded with 50 µl 1 M Tris.HCl pH 8.0 for neutralization) and diafiltrated against PBS pH 7.2. Dynabeads coated with FCR3 recombinant DBL5-6-ε was used to deplete goat IgG from anti DBL5-6-ε antibodies. 100 µg of recombinant FCR3-DBL5-6-ε were coated on 166 µl washed Dynabeads M-280 tosylactivated (Invitrogen Dynal, Oslo, Norway) prepared according to manufacturer's indications. Aliquots of 166 µl of beads were washed three times in 900 µl PBS pH 7.4 using the magnetic particle concentrator (Dynal) and resuspended in the same buffer. The washed beads were mixed with 100 µg (1 mg/ml) of protein, 1.2 M ammonium sulfate and incubated 24 h at 37°C with slow tilting. After incubation beads were washed five times with 1 ml PBS, incubated 1 h at 37°C with PBS, 0.5% BSA and washed 2 times in PBS. The beads were stored in same buffer. 100 µl of purified IgG at 5 mg/ml were incubated during one hour with the coated beads. The flow through was used for flow based inhibition assay. The coated magnetic beads were washed four times with 1 ml PBS and the antibodies were eluted with 0,1 M glycine pH 2.5. Eluted fractions were neutralized with Tris.HCl 1 M pH 8.0. Pre-immune goat IgG was depleted in the same condition and used as control.

### 4. Cell Culture

The FCR3 strain was grown in RPMI 1640 supplemented with bicarbonate, glutamine, 0.2% glucose, 50 µM hypoxanthine, 10 µg/ml gentamicin, 25 mM HEPES and 0.25% Albumax containing erythrocytes in a candle jar at 37°C [19]. Clone ScC2 Saimiri brain microvascular endothelial [20] and the human choriocarcinoma-derived cell line BeWo (ATCC Nr CCL98) were grown in DMEM/F12 (Sigma) supplemented with bicarbonate, glutamine, 10 µg/ml gentamicin, 10% fœtal calf serum (Gibco) and 15 µg/l endothelial cell growth supplement (Sigma). The cells were grown in a humidified atmosphere in 5% CO2.

### 5. Flow-based Cytoadhesion Assay

ScC2 or BeWo cell suspensions were grown on Slideflask (Nunc DK) to confluence. The slides were then mounted in a cell adhesion flow chamber CAF 10 (Immunetics, Boston MA, USA) and held in place by vacuum. The system was connected to a precise perfusion peristaltic pump P720 (Instech laboratories Inc, Winsum, NL). The outlet of the perfusion chamber was connected to reservoirs containing PE suspension in cytoadhesion medium (RPMI, pH = 6,8) [21]. Cytoadhesion was observed with an inverted microscope (Nikon Eclipse TE200, Japan) using Plan Apo 10/0,45 objective (Nikon) coupling to camera and Lucia 4.8 software (Nikon) for the numeration. For the recombinant protein inhibition assays, slides were preincubated with the indicated quantities diluted in cytoadhesion medium for 1 h at 37°C. An aliquot of 1 ml of PE suspension at 2×10^7^/ml incubated 15 min at 37°C with purified antibodies, CSA (Sigma), or only medium, was allowed to flow into the perfusion chamber over the ScC2 or BeWo at 0.05 Pa. We then allowed cytoadhesion medium to flow through the chamber for 10 min at 0.05 Pa to remove non-adherent cells. The number of adherent PE/mm^2^ was determined in two to three independent experiments.

### 6. Flow Cytometry

Trophozoite-stage PEs were purified using gelatin flotation and incubated with mice or goat sera that had been preabsorbed twice with uninfected erythrocytes. For each assay, 10 million PEs were incubated with a 1/10 dilution of sera. Bound antibodies were detected by adding biotin-conjugated goat anti-mouse IgG (Sigma, Saint Louis, MO) or biotin-conjugated rabbit anti-goat (ab7131, Abcam) followed by fluorescein isothiocyanate (FITC)-conjugated streptavidine (BD pharmingen, San Diego, CA). Analysis was carried out by gating parasitized cells stained with 10 µg/ml of ethidium bromide (Eurobio, France) on a FACScan using CellQuest software (BD Biosciences). At least 50000 cells were counted for each sample, in two independent experiments.

## Results

### Expression and purification of recombinant DBL5-ε, DBL6-ε and DBL5-6-ε domains

Due to the large protein size, var2CSA-based vaccine development is highly dependent on the production of functional var2CSA protein subunits. Our previous analysis of mouse mAbs directed against the surface of FCR3^CSA^ suggested that the C-terminal region (DBL5-6-ε) of var2CSA was frequently recognized by adhesion blocking mAbs [22]. For this reason, we focused our effort on DBL domains DBL5-ε, DBL6-ε, and double DBL5-6-ε domains. These domains were cloned into the pET28a vector (Novagen, His-tag on the N-terminus) for subsequent expression and purification. We expressed DBL5-ε domains from FCR3, Dd2, HB3, and 7G8 parasite strains, and DBL5-6-ε domain from FCR3 and HB3. Only FCR3-DBL6-ε was expressed as a soluble protein, whereas DBL5-ε and DBL5-6-ε accumulate in inclusion bodies as insoluble aggregates.

**Figure 1:**
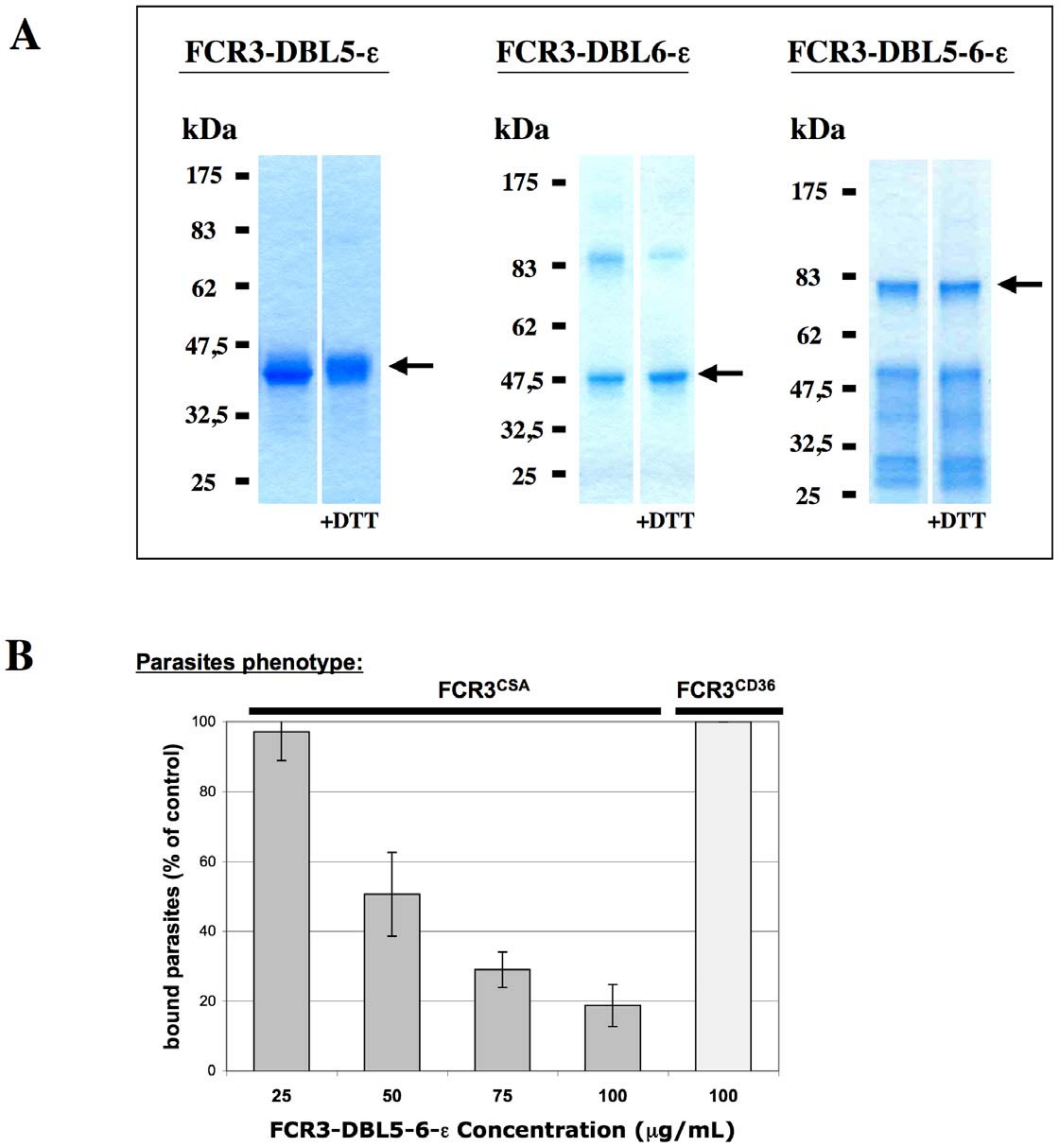
Expression and purification of recombinant var2CSA domains. **(A)** Purification after refolding of FCR3 var2CSA DBL domains expressed in *E. coli*. Electrophoresis of purified DBL domains under reduced (+DTT) or non-reduced conditions on NuPAGE Novex 4–12% Bis-Tris gels, Coomasie bleu stained. Arrows indicate the expected proteins. **(B)** FCR3-DBL5-6-ε recombinant domain competes the adhesion of var2CSA expressing FCR3-PEs but fails to inhibit binding of FCR3^CD36^ parasites on CD36 receptor. The adhesion of FCR3-PEs was tested in flow binding assays using ScC2 cells. FCR3-DBL5-6-ε recombinant protein inhibited FCR3^CSA^ parasite binding in a phenotype specific and dose-dependent manner.

FCR3-DBL6-ε was purified by His-tag affinity from the supernatant obtained after lysis of bacteria and centrifugation (Figure 1A). Highly pure (over 95%) recombinant protein was obtained at a yield of 2.5 mg/liter of culture. No further purifications steps were necessary. Western blot analysis showed that the larger band around 90 kDa corresponds to a dimer of DBL6-ε (data not shown).

Inclusion body containing DBL5-ε and DBL5-6-ε domains were solubilized in a 7 M Guanidine.HCl buffer and refolding tests were carried out by the method of rapid dilution. Using this strategy, the best solutions for refolding of each insoluble domain were chosen according to relative criteria of yield, solubility and his-tag affinity. After affinity purification all proteins solutions were dialysed against PBS. Several bands were observed for FCR3-DBL5-6-ε on a SDS-PAGE (Figure 1A). N-terminal sequencing and western blot using anti-His-tag suggest that these proteins are degradation products derived from FCR3-DBL5-6-ε. In our hands shift in gel migration of cysteine-rich proteins in the presence of DTT as reducing agent were not systematically observed. A slight shift in migration for FCR3-DBL5-ε can be observed in Figure 1A. The absence of shift for FCR3-DBL6-ε was already observed for this domain when expressed in an eukaryotic system (HEK293 Cells) where these disulfide bridges are normally produced [16].

In a previous study, we showed that DBL6-ε expressed on the surface of CHO cells binds to CSA-coated beads [11]. The recombinant var2CSA FCR3-DBL5-6-ε purified protein was capable to compete in a dose-dependent manner the binding of FCR3^CSA^ PEs to ScC2 cells while not affecting the FCR3^CD36^ PEs (Figure 1B). This result indicates that the refolded recombinant protein is in a functional conformation. HB3-DBL5-6-ε showed similar results when expressed, refolded and purified using the same conditions as for FCR3-DBL5-6-ε (data not shown).

### Anti DBL5-ε, DBL6-ε, and DBL5-6-ε antibodies inhibit adhesion to BeWo cells under flow conditions

To investigate whether the recombinant var2CSA domains could induce biologically active antibodies, groups of five mice were immunized with FCR3-DBL6-ε and FCR3-DBL5-6-ε recombinant proteins. Two different adjuvants were compared in this test: Freund and Alum. Final bleed antisera were pooled for each group, IgG purified and evaluated for their capacity to recognize the surface of FCR3^CSA^ binding PEs by liquid immunofluorescence. All IgG pools showed surface reactivity with CSA-panned but not with CD36 panned parasites (data not shown).

**Figure 2:**
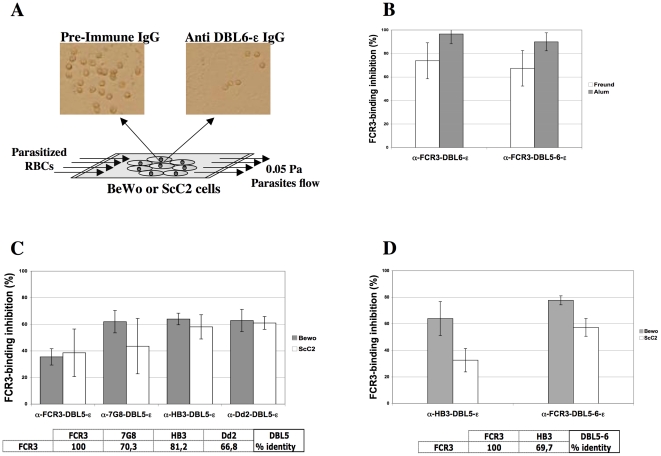
Recombinant DBL5-ε, DBL6-ε, and DBL5-6-ε domains induce antibodies that block adhesion to BeWo cells in flow binding assays. **(A)** Schematic representation of the binding inhibition assay mimicking the flow in placental physiological conditions **(B)** Inhibition of FCR3 PEs adhesion. Purified IgG at 0.5 mg/ml from a group of 5 mice immunized either with Freund or Alum as adjuvants were tested for inhibition of binding of FCR3-PEs to BeWo cells in flow binding assays. The numbers of bound PEs in the presence of IgG from preimmune serum was used as a reference value. The numbers of bound PEs for anti DBL IgG were used to calculate the binding inhibition as a percentage relative to the reference value. **(C)** and **(D)** Recombinant domains from heterologous parasites induce transcending adhesion blocking activity. Purified IgG at 0.5 mg/ml from groups of 5 mice immunized with heterologous DBL5-ε and DBL5-6-ε domains were tested for inhibition of binding of FCR3-PEs to BeWo and ScC2 cells in flow binding assays. Amino acid sequence identity percentages between the heterologous DBL domain and the homologous FCR3 DBL are presented.

The ability of the immune sera IgG to inhibit the binding of FCR3^CSA^ PEs to the CSA expressing human placental derived BeWo cell line [23] was assessed in a flow-based cytoadhesion assay mimicking the physiological blood circulation in the human placenta (Figure 2A). Mouse preimmune sera IgG were tested to exclude non-specific inhibition. DBL6-ε and DBL5-6-ε antisera IgG inhibited binding of FCR3^CSA^ PEs to BeWo cells with values of 67.4% and 73.8% for Freund's group and 89.9% and 96.6% for Alum's group, respectively (Figure 2B). FCR3-DBL5-ε antisera IgG obtained with Freund as adjuvant inhibited binding of FCR3^CSA^ PEs to BeWo cells at 35.5% (Figure 2C).

### Antibodies against heterologous DBL5-ε and DBL5-6-ε crossreact with FCR3^CSA^ binding strain

Given the promising results obtained with Alum, all new immunizations were done with this adjuvant. Groups of five mice were immunized with Dd2-DBL5-ε, HB3-DBL5-ε, 7G8-DBL5-ε, and HB3-DBL5-6-ε recombinant antigens. The ability of the antisera IgG to inhibit the binding of FCR3^CSA^ PEs to the CSA expressing human placental derived BeWo cell line [23] and the endothelial cell line ScC2 [24] was assessed using the flow-adhesion system. All IgG pools inhibited binding of FCR3^CSA^ PEs when compared to the preimmune sera IgG with values ranging from 32.7% to 77.6% (Figure 2C and 2D). It is noteworthy, that inhibition on BeWo cells gave slightly higher inhibition levels when compared to monkey brain endothelial cell line ScC2 expressing CSA.

### DBL5-6-ε elicits cytoadhesion blocking antibodies in goat that can be depleted with recombinant DBL5-6-ε protein

**Figure 3:**
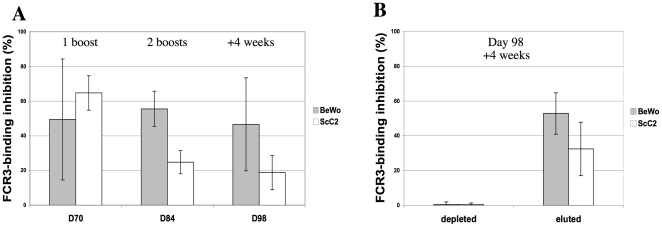
Recombinant FCR3-DBL5-6-ε depletes the adhesion blocking activity in goat serum. **(A)** Goat Immunization with FCR3-DBL5-6-ε induces adhesion blocking antibodies. Goat purified IgG was tested for inhibition of FCR3-binding of PEs to BeWo and ScC2 cells in flow binding assays after the first boost, second boost and four weeks after the second boost. **(B)** Recombinant FCR3-DBL5-6-ε depletes the adhesion blocking activity. Goat purified IgG obtained four weeks after the second boost (D98) was depleted on immobilized FCR3-DBL5-6-ε. The inhibition of FCR3-PEs adhesion of IgG before antigen depletion, the depleted IgG, and elution of anti DBL5-6-ε were tested in flow binding assays using BeWo and ScC2 cells.

While mice offer the advantage of working in a consistent genetic background, are easy to handle, and are very cost-effective, they provide limited quantities of serum for IgG characterization under flow adhesion. We therefore immunized a goat with var2CSA FCR3-DBL5-6-ε recombinant domain and tested the produced antisera over time for their ability to recognize the native var2CSA in FCR3^CSA^ PEs by liquid immunofluorescence and their capacity to inhibit the binding of FCR3^CSA^ PEs to BeWo and ScC2 cells. One goat was immunized with 200 µg of var2CSA FCR3-DBL5-6-ε recombinant domain using Alum as adjuvant, followed by boosts of equal amounts of antigen at day 35 and day 70. Samples were taken five weeks after the first boost (D70) and two (D84), and four (D98) weeks after the second boost. The IgG purified from sera was able to recognize specifically FCR3^CSA^ PEs by liquid immunofluorescence (data not shown), and inhibited the binding to BeWo (approx. 50%) and ScC2 cells (64 to 18%) (Figure 3A). To investigate if this binding inhibition was specific to anti DBL5-6-ε antibodies, we immobilized the FCR3-DBL5-6-ε recombinant antigen in tosylactivated beads and adsorbed a fraction of D98 goat antisera IgG. When measuring binding under flow conditions the IgG sample flow through lost its binding inhibition capacity. Elution of retained IgG allowed the recovery of inhibition ability to similar levels as before treatment (Figure 3B).

### Antibodies against homologous and heterologous DBL5-ε and DBL5-6-ε domains react differentially with the surface of FCR3 PEs

**Figure 4:**
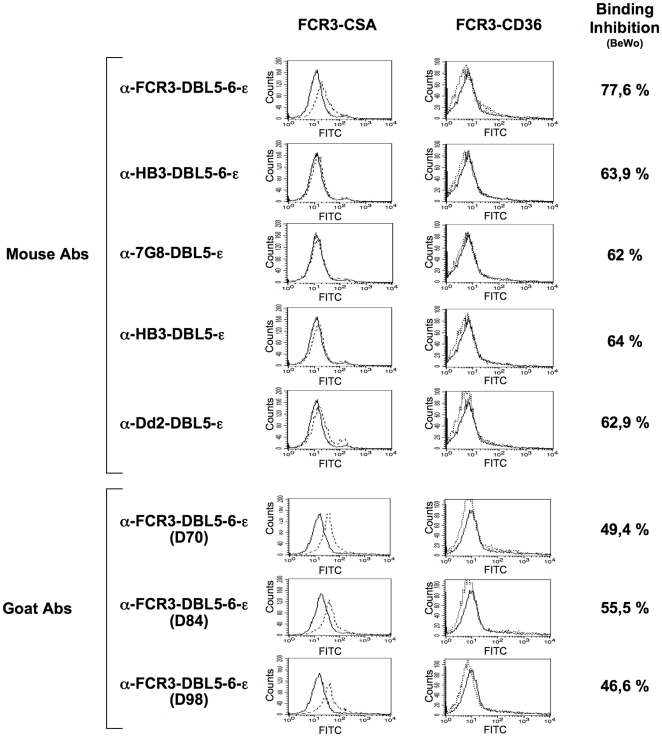
Recognition of antigens expressed on the surface of PEs. Mouse and goat sera raised against DBL domains were assayed for their binding capacity to the surface of erythrocytoes infected with parasites of the FCR3 strain with CSA- and CD36-binding phenotypes. Analysis was performed using flow cytometry. Panels represent number of events (counts) against fluorescence (FITC) for each antiserum (doted line) when compared to the pre-immune serum (full line). FCR3^CSA^ parasite binding inhibition on BeWo cells are indicated for the respective antisera.

In order to investigate if surface reactivity with FCR3 PEs could be predictive for inhibition of adhesion to the placenta, we measured the reactivity of our mouse and goat antisera with live PEs using flow cytometry. The results are shown in figure 4. All sera directed against the FCR3-DBL5-6-ε domains reacted strongly with the homologous FCR3 parasite at serum dilutions 1/10. The anti FCR3-DBL5-ε antibodies had been used up for inhibition studies. The antibodies raised against DBL5-ε and DBL5-6-ε from heterologous parasites (HB3, 7G8 and Dd2) showed a weak surface reactivity with FCR3, suggesting that a large portion of the surface antibodies is allele specific. Nonetheless, the binding inhibition of these heterologous antibodies was generally >60% using FCR3^CSA^ parasites. This result obtained with anti recombinant DBL antisera has important implications since it shows that surface reactivity is not predictive for binding inhibition.

## Discussion

In this work we showed that recombinant DBL domains corresponding to the C-terminal var2CSA external region generate strong cytoadhesion-blocking antibodies (>50% inhibition at 0.5 mg/ml) in different experimental animals. Furthermore, we also show that the inhibitory antibodies are specifically targeting the antigen as the inhibition capacity is lost after depletion of the antisera with the recombinant protein.

The use of VAR2CSA as a vaccine candidate to prevent PAM is hampered by its high molecular weight together with its hydrophobic and cysteine-rich sequence as major restrictions for production of sufficient functional full-length recombinant protein. Although recent reports show that the expression of the entire external domain of var2CSA is now possible [12], [13], its use for vaccine development is obviously limited due to the constraints in up-scaling production. Several epitopes have been described in var2CSA, which induce cytoadhesion-blocking antibodies [16]–[18] . A major research effort is currently dedicated to explore the degree of cross-reactivity with other alleles.

DBL5 is one of the most conserved var2CSA regions, its allelic forms vary between 66% for Dd2 to 81% for HB3 when compared to the reference strain FCR3. Immunization with three heterologous DBL5 allelic forms generated cytoadhesion-blocking antibodies to the reference strain FCR3. Importantly, this transcending biological activity has been obtained in an assay system that mimics closely placental flow of parasites. Our results are not totally in line with already published data using different expression systems. For example DBL5-ε expressed in *Pichia pastoris* induces highly cross-reactive antibodies but with limited or no inhibitory activity [25]. In addition only very limited parasite inhibition using DBL5-specific serum was obtained in another laboratory (DBL5-ε produced in the baculovirus expression system) [18]. The observed variation in adhesion blocking antibodies between laboratories may be the result of DBLs generated in different protein expression systems, which may produce slightly different DBL conformations. It is also possible that antibody mediated desequestration is more easy on BeWo cells than on plastic coated CSA. This is the first study which analyzes sera from PAM vaccine candidates in a flow assay using placental trophoblastic cell line. Our data are very promising and point to DBL5 as a subunit vaccine with a high desequestration potential. The next important step will be to evaluate the efficacy of inhibition of placental isolates from distinct geographic regions.

In conclusion, our results clearly show that a single DBL domain produced in *E. coli* can achieve significant levels of parasite cytoadhesion inhibition on placental cells. Given the transcending immune response against the relatively conserved DBL5 domain this antigen is likely to inhibit sequestration of infected red blood cells in the placenta. The fact that the immunogenicity is potentiated by the use of Alum, an adjuvant used in humans is very promising for next steps. At this stage, we cannot exclude that the development of an efficacious subunit-based recombinant vaccine against PAM might require a cocktail of subunit antigens. Further studies on clinical isolates are necessary to validate our findings.
